# Using an Improved SIFT Algorithm and Fuzzy Closed-Loop Control Strategy for Object Recognition in Cluttered Scenes

**DOI:** 10.1371/journal.pone.0116323

**Published:** 2015-02-25

**Authors:** Haitao Nie, Kehui Long, Jun Ma, Dan Yue, Jinguo Liu

**Affiliations:** 1 Changchun Institute of Optics, Fine Mechanics and Physics, Chinese Academy of Sciences, Changchun, China; 2 University of Chinese Academy of Sciences, Beijing, China; Peking University, CHINA

## Abstract

Partial occlusions, large pose variations, and extreme ambient illumination conditions generally cause the performance degradation of object recognition systems. Therefore, this paper presents a novel approach for fast and robust object recognition in cluttered scenes based on an improved scale invariant feature transform (SIFT) algorithm and a fuzzy closed-loop control method. First, a fast SIFT algorithm is proposed by classifying SIFT features into several clusters based on several attributes computed from the sub-orientation histogram (SOH), in the feature matching phase only features that share nearly the same corresponding attributes are compared. Second, a feature matching step is performed following a prioritized order based on the scale factor, which is calculated between the object image and the target object image, guaranteeing robust feature matching. Finally, a fuzzy closed-loop control strategy is applied to increase the accuracy of the object recognition and is essential for autonomous object manipulation process. Compared to the original SIFT algorithm for object recognition, the result of the proposed method shows that the number of SIFT features extracted from an object has a significant increase, and the computing speed of the object recognition processes increases by more than 40%. The experimental results confirmed that the proposed method performs effectively and accurately in cluttered scenes.

## Introduction

Object recognition has become one of the most active research topics in the fields of computer vision and pattern recognition because of its potential value in practical applications. Many novel methods have been proposed in the literature for object recognition, and they can be broadly classified into two main categories: holistic methods and local feature-based methods. The holistic methods attempt to recognize the object as a whole. Thus, the query image is acquired, pre-processed, and segmented, and the global features are extracted. Finally, statistical classification techniques are used. This class of algorithms is especially suited to homogeneous objects, which can be easily segmented. Typical holistic methods can be found in [1–4]. The holistic methods are simple and fast, but there are limitations in recognition during changes in illumination and poses. Local feature-based methods, however, are better suited to textured objects and are more robust with respect to variations in viewpoint and illumination. Local feature-based methods are based on the idea of representing an object by a collection of local invariant patches. Generally, local feature-based methods primarily involve the following steps: first, salient points, which are typically corners or blob-like shapes from the image to be matched, are extracted. Second, descriptors from regions around the salient key-points are constructed using mechanisms that aim to keep the characteristics of these regions insensitive to viewpoint and illumination changes and invariant to rotation, scaling and affine transformations. Finally, correspondence points between the query and model images are computed based on extracted features. From the matched points, an affine transformation between the query and model images can be computed using a fitting method, such as Least of Squares and random sample consensus (RANSAC) method [5]. The matching process is then iteratively refined by removing the correspondence points that do not fit this affine transformation. The idea originates from the work of Schmid and Mohr [6], whereby the centers of patches are located at points of interest and are invariant under rotation. Typical local feature-based methods can be found in [7–10]. Lowe developed an efficient object recognition approach based on the scale invariant feature transform (SIFT) [11].

The SIFT algorithm, proposed by Lowe, is one of the most widely used local feature-based method for object recognition and is useful for nearly all computer vision tasks. The algorithm attempts to detect similar feature points in each of the available images and subsequently describe these points with a feature vector, which is invariant to scale and rotation and is partially invariant to illumination and viewpoint changes. In addition to these properties, SIFT features are highly distinctive and relatively easy to extract and match against large databases of local features. However, the main drawback of the SIFT algorithm is that the computational complexity of the algorithm increases rapidly with an increasing number of key points, especially during the matching step due to the high dimensionality of the SIFT feature descriptor. To overcome the main drawbacks of the SIFT algorithm, various modifications have been proposed. In general, strategies addressing the acceleration of SIFT feature matching can be classified into three different categories: reducing the descriptor dimensionality [12], [13], using parallelization and exploiting the power of hardware [14–16], and improving feature matching algorithms [17–19].

Traditional local feature-based object recognition methods are open-loop methods, which mean that the result of each step depends on the result of the previous step. Therefore, errors are accumulated over the entire recognition system and propagated to the final step. Hence, the final result tends to be error prone and unreliable. This problem is usually solved using closed-loop control techniques. Because the method is non-linear and no mathematical model is available, a fuzzy control strategy is used. Ever since fuzzy set theory was used to synthesize a fuzzy logic controller for a simple dynamic process in Mamdani and Assilian [20], fuzzy logic control has become one of the most successful applications of the theory. An important application of a fuzzy-knowledge-based system is the control of complex, nonlinear systems [21]. Control algorithms with fuzzy controllers offer better response and efficiency in the case of complex nonlinear systems when compared to conventional controllers [22], [23]. The basic difference between fuzzy and conventional controllers is that the latter are designed using a mathematical model for the process being controlled. In contrast, fuzzy controllers are based on the synthesis of prior knowledge, which is provided by human expertise to construct a set of rules in the form of IF-THEN statements [24]. Typically, the design of a fuzzy controller is mostly based on expert control experience [25] or on a self-learning process [26], which requires human experience to design fuzzy control systems that demonstrate good performance.

In this paper, we propose an improved SIFT algorithm and a fuzzy closed-loop control strategy for object recognition in cluttered scenes. A fast SIFT algorithm is proposed by classifying SIFT features into several clusters based on several attributes computed from the SIFT orientation histogram, in the feature matching step, only features that share nearly the same corresponding attributes are compared. Feature matching is performed following a prioritized order based on the scale factor, which is calculated between the object image and target image to guaranteeing robust feature matching. A fuzzy closed-loop control strategy based on SIFT features is applied to increase the invariant to affinity, thereby increase the quality of the results of the matching process, which is essential for autonomous object manipulation. To compare the improved SIFT algorithm with the original SIFT algorithm, the proposed method was compared with two algorithms for approximate nearest neighbors (ANN) searching, hierarchical k-means tree (HKMT) [27] and randomized KD-trees (RKDTs) [28]. The hierarchical k-means tree is constructed by splitting the data points at each level into K distinct regions using k-means clustering, and then applying the same method recursively to the points in each region. We stop the recursion when the number of points in a region is less than K. The randomized KD-trees are built by choosing the split dimension randomly from the first D dimensions on which the data have the greatest variance. When searching the trees, a single priority queue is maintained across all the randomized trees such that the search can be ordered by increasing distance to each bin boundary. The degree of approximation is determined by examining a fixed number of leaf nodes, at which point the search is terminated and the best candidates returned. The presented experimental results indicate that the proposed method outperforms the two other considered algorithms. Additionally, several images from a stranded image database and from real-world stereo images were tested under different conditions, in which viewpoint were altered, partial occlusion, pose invariant, or the illumination during image acquisition conditions. The experimental results confirmed that the proposed method performs effectively and accurately for object recognition in cluttered scenes.

## Materials and Methods

### Fast SIFT algorithm

**Fig 1:**
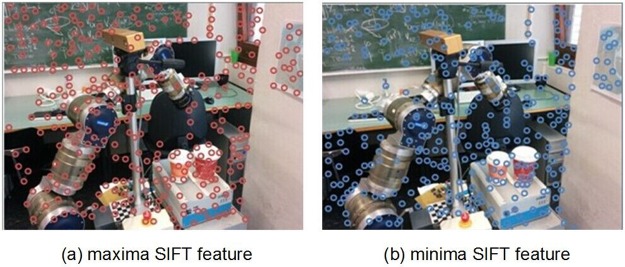
The Maxima and Minima SIFT features extracted from the same image.

**Fig 2:**
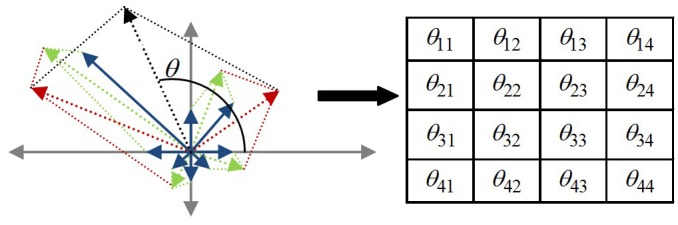
Angles computed from the sub-orientation histogram.

Although there have been early impressive object recognition results achieved using the SIFT algorithm, efficient object recognition under cluttered-scene conditions is still challenging. To achieve fast object recognition a novel strategy to accelerate the SIFT features-matching process is introduced in this paper. The strategy is based on hashing of SIFT features into several clusters during the feature extraction phase using new attributes computed from the SOH. First, in the key point detection stage, the key points are split into two types: Maxima and Minima. Fig. 1 presents the Maxima and the Minima SIFT features extracted from the same image. The Maxima SIFT feature locations are centers of dark blobs on a light background and vice versa for the Minima locations. The number of Maxima SIFT features is nearly equal to the number of Minima SIFT features extracted from the same image. Only the features of the same type are computed, and the matching time is reduced by 50% with respect to the exhaustive search without losing any correct matches. No matches can be expected between two features of different types. Second, SIFT features are extended by a few new angles without incurring extra computational costs. The high dimensionality of the SIFT descriptor (SIFT-D) makes feature matching very time consuming. To speed up the feature matching process, it is assumed that some new independent angles can be assigned to each feature. These angles are invariant to changes in the viewing geometry and illumination, and they are computed from sub-orientation histograms (SOHs) of the SIFT-D. In the original SIFT algorithm for computation of the SIFT-D, the interest region around the key point is subdivided into sub-regions in a rectangular grid. From each sub-region, an SOH is built [29]. Theoretically, a SIFT feature can be extended using a number of angles equal to the number of SOHs because these angles are to be calculated from the SOHs. In the case of 4x4 grids, the number of angles is 16, as shown in Fig. 2.

However, to speed up SIFT matching, these angles should be components of a multivariate random variable that is uniformly distributed in the 16-dimensional space [-180°, 180°]. To meet this requirement, the following two conditions must be verified. First, each angle must be uniformly distributed in [-180°, 180°] [30]. Second, the angles should be pair-wise independent [31]. The angles between the orientations corresponding to the vector sum of all bins of each SOH and the horizontal orientation are suggested as the SIFT feature angles. Mathematically, the proposed angles {*ϴ*_*ij*_; i, j = 1,..,4} are calculated as follows:

$$\theta_{\text{ij}}\text{=}\tan^{-1}\left(\frac{\sum_{k=0}^{7}mag_{ij}(\text{k})\cdot \text{sin}(\text{or}{\text{i}}_{ij}(\text{k}))}{\sum_{k=0}^{7}mag_{ij}(\text{k})\cdot \cos(\text{or}{\text{i}}_{ij}(\text{k}))}\right)$$ (1)

where *mag*(*k*) and *ori*(*k*) are the magnitude and orientation of the *k* ^*th*^ bin of the *ij* ^*th*^ SOH, respectively. Because the angles *ϴ*_*ij*_ are computed from SOHs from which the SIFT-D is built, they are invariant under geometrical and photometrical transformations. Finally, four angles can be pair-wise independent, and only border angles can meet the equally likely condition. Therefore, the best choices are the corner angles, *ϕ*_1_ = *θ*_11_, *ϕ*_2_ = *θ*_14_, *ϕ*_3_ = *θ*_41_ and *ϕ*_4_ = *θ44*, which can be considered as new attributes of the SIFT feature. Compared with the original 128-dimensional SIFT descriptor, the improved SIFT algorithm can lead to a significant decrease in computational time.

**Fig 3:**
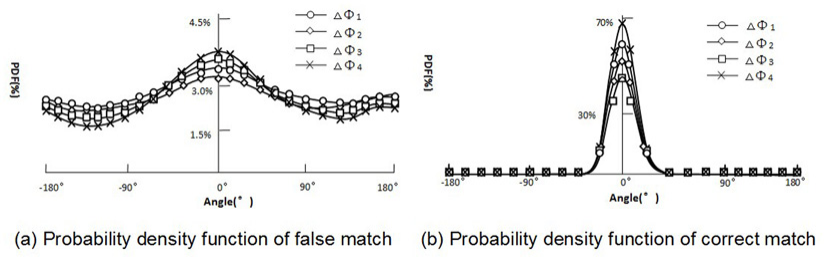
Probability density function of the angle differences.

At the matching stage, a new idea is proposed based on the new angles by comparing features that share the same corresponding angles, which may lead to correct matches. Among all possible matches, only a small number of correct matches exist. For each possible match, four different angle differences {Δ*ϕ*_11_, Δ*ϕ*_22_, Δ*ϕ*_33_, Δ*ϕ*_44_} for each pair of SIFT features can be constructed. Considering the angle differences as random variables, the behaviors of these random variables vary according to the type of matches being analyzed. Fig. 3 shows the probability density function of the angle differences. For a false match, its feature is independent. Therefore, each of the two corresponding angles is independent because four random variables are uniformly distributed and are pair-wise independent. However, each of the corresponding correct match angles tends to be equal because the features of the correct matches tend to have the same SIFT descriptors. Therefore, the four random variables tend to be concentrated at approximately 0°. By calculating the probability density function of the random variables, approximately 95% of the correct matches and only 15% of the false matches are found to belong to the range ${\left[\text{-}36^{0},36^{0}\right]}^{4}$. Because the possible matches are uniformly distributed in the 4-dimensional angle space ${\left[\text{-}180^{0},180^{0}\right]}^{4}$, the portion of possible matches in the range ${\left[\text{-}36^{0},36^{0}\right]}^{4}$ is equal to (72360)4·100%. To exploit this outcome, SIFT features are hashed into a 4-dimensional table based on their angles. The SIFT features of each cell are compared only with the features of some cells such that the correspondences have absolute difference in angle that are less than a pre-set threshold. A threshold of 36° was selected because almost all correct matches have angle differences in the range ${\left[\text{-}36^{0},36^{0}\right]}^{4}$.

Thus far, we have extended a SIFT feature by adding 4 pair-wise-independent angles that are invariant to rotation, scale and illumination changes. During the extraction phase, the SIFT features are classified based on their angles into different clusters. Thus, in the matching phase, only SIFT features that belong to clusters from which correct matches may be expected are compared. A fast SIFT algorithm is concluded to confirm the efficiency of object recognition system.

### Robust SIFT feature matching

Although SIFT features are reasonably invariant, they cannot accommodate large changes in viewpoint or extreme illumination conditions, which is the core problem of object recognition in cluttered scenes. This problem is caused by the absence of true positive correspondences or by their portion being insufficient for fitting methods to work correctly. This paper introduces a new procedure to determine the scale factor between object images to be recognized by dividing SIFT features into different sub-sets based on their octaves. Then, the matching process is performed following a prioritized order, whereby only features of the same scale ratio are compared in each step. Additionally, a scale ratio histogram (SRH) is constructed. Only matches of the step corresponding to the highest SRH bin are provided to the fitting method. This restriction decreases the portion of outliers among positive matches, leading to an improved performance of the fitting method, which is called a random sample consensus. This strategy exhibits an increased matching performance and robustness with no additional computational time cost.

**Fig 4:**
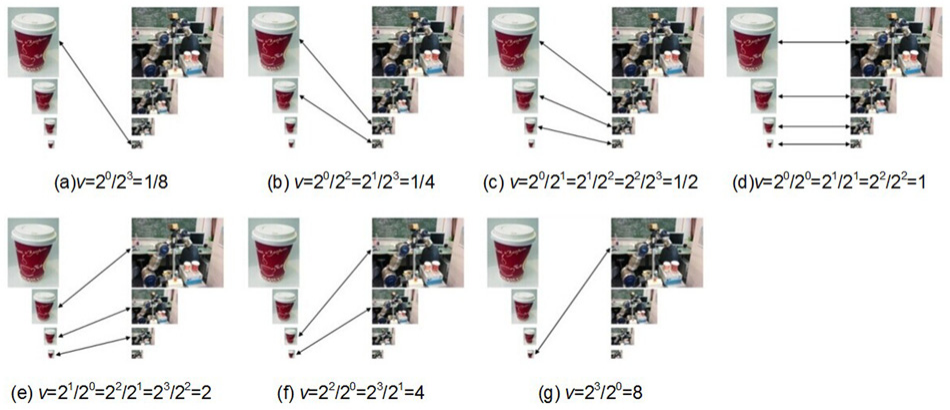
The steps of the calculation scale factor.

In general, the SIFT algorithm is a local image operator that takes an input image and transforms it into a collection of local features. To use the SIFT operator for object recognition purposes, it is applied on two images: a model image and test image. The model image presents only the object taken under predefined conditions, whereas the test image is an image including the target object captured in cluttered scenes. Using the SIFT operator, the two object images are transformed into two SIFT image feature sets. These two features sets are divided into subsets according to the octaves in which the features arise. To perform the newly proposed SIFT feature matching strategy, the feature subsets obtained are arranged so that a subset of the model image feature set is aligned to a subset of the test image feature set. The process of aligning the model image subset with the test image subset is performed in n+m-1 steps, where n and m are the total number of octaves (subsets) corresponding to the model image and test image, respectively. For each step, all pairs of aligned subsets must have the same ratio v, which is defined as:

v=2o12o2 (2)

where *o*_1_ and *o*_2_ are the octaves of the model image subset and the test image subset, respectively. For example, for both the model and test images, if there are four octaves, the process will consist of seven steps. At the first step *a*, only SIFT features of the model image extracted from octave *o*_1_ = 0 are compared with the SIFT features of the test image extracted from octave *o*_2_ = 3. In this case, all possible matches have the scale ratio v=2023=1/8. In step *b*, only the model SIFT features of the octaves *o*_1_ = 0 and *o*_1_ = 1 are compared with the test SIFT features of the octaves *o*_2_ = 2 and *o*_2_ = 3, respectively. In both cases, the possible matches have a scale ratio of v=2022=2123=1/4 for other steps, as indicated by the arrows in Fig. 4. At every step, the total number of positive matches is determined for each aligned subset pair. The total number of positive matches within each step is indexed using the appropriate shift index:

$$k=o_{1}-o_{2}$$ (3)

The shift index can be negative, positive or zero. The highest number of positive matches achieved determines the optimal shift index *k*_*opt*_ and the consequent scale factor:

$$S=2^{k_{opt}}$$ (4)

To realize the proposed procedure mathematically, a scale ratio histogram F(x) is defined as

$$\text{F}\left(x\right)=\left\{ \begin{array}{l} \sum_{j=0}^{x}R\left(M_{1}^{n-1-x+j},M_{2}^{j}\right)x<\text{n}\\ \sum_{i=0}^{j=n-1}R\left(M_{1}^{n-1-x+j},M_{2}^{j}\right)n\leq x<m\\ \sum_{i=0}^{j=m+n-2-x}R\left(M_{1}^{x\text{-}n+j},M_{2}^{x-m+1+j}\right)x\geq m \end{array}\right.$$ (5)

where $R(M_{1}^{i},M_{2}^{j})$ is the number of positive matches between the *i* ^t*h*^ subset of the model image feature set $M_{1}^{i}$ and the *j* ^t*h*^ subset of the test image feature set $M_{2}^{j}$ and *x* is the modified shift index introduced for the sake of simplicity in the above equation.

$$x=\mathrm{int}(k+(\frac{n+m-1}{2}))$$ (6)

The scale ratio histogram *F*(*k*) obtains its maximum at the shift index *K*_*opt*_ = *arc* max (*F (K*)) = 1,which corresponds to the scale factor.*S* = 2^*kopt*^ The optimal shift index defines a “domain of correct matches”. All matches outside this domain, including positive matches, are excluded. The positive matches from the domain of correct matches are used to determine the affine transformation (rotation, matrix, and translation vector) between the two feature sets using the RANSAC method. Once the transformation is calculated, every match that is either positive or negative within the domain of correct matches is examined to determine if it meets the previously calculated transformation. If the match fulfills the transformation, it is labeled as correct; otherwise, it is labeled as a false match. Thus, this method can significantly reduce the number of false match features.

**Fig 5:**
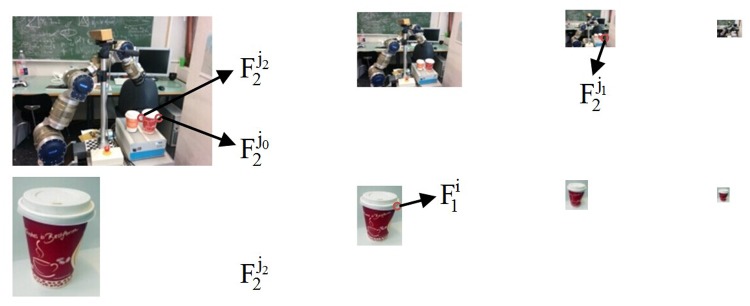
Searching the second nearest feature in the limiting area.

Among all the found matches, many correct matches will exceed Lowe’s threshold τ. To retrieve these correct matches, the ratio between the Euclidean distance to the nearest feature neighbor and the Euclidean distance to the second nearest feature neighbor must be reduced. The feature $F_{1}^{i}$ from the model image feature set is correctly assigned to the feature $F_{2}^{j_{0}}$ from the test image feature set. Additionally, suppose that $F_{2}^{j_{1}}$ is the second nearest feature to feature $F_{1}^{i}$. Reducing the ratio can be performed by either reducing the smallest distance $d_{1}(F_{1}^{i},F_{2}^{j_{0}})$ or by increasing the next smallest distance $d_{2}(F_{1}^{i},F_{2}^{j_{1}})$. In practice, the first alternative is impossible, whereas the enlargement of the next smallest distance can be achieved by limiting the search area for both the nearest feature and the next nearest feature to the feature $F_{1}^{i}$ within a specified domain. Feature $F_{2}^{j_{2}}$ is the second nearest feature to $F_{1}^{i}$ when the search is limited only to the octave in which the features $F_{2}^{j_{0}}$ are found. As shown in Fig. 5, because the distance $d_{3}(F_{1}^{i},F_{2}^{j_{2}})\;\geq \;d_{2}(F_{1}^{i},F_{2}^{j_{1}})$ always holds, the following is obtained:

d1(F1i,F2j0)d2(F1i,F2j1)≥d1(F1i,F2j0)d3(F1i,F2j2) (7)

Thus, by reducing the search area, the ratio related to the feature $F_{1}^{i}$ can be decreased and can be less than the threshold τ. The proposed method improves the object feature matching robustness of object recognition in a cluttered background.

### A fuzzy closed-loop control strategy for object recognition based on SIFT features

**Fig 6:**
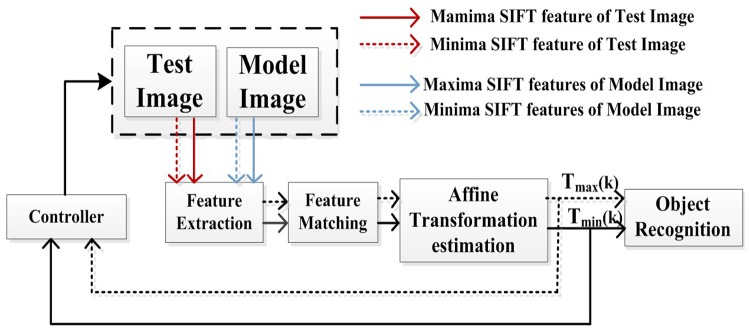
Proposed fuzzy closed-loop object recognition system.

A fuzzy closed-loop control approach is proposed for object recognition based on SIFT features. This approach uses the benefits of fuzzy closed-loop structure to increase the invariance to affinity, and consequently, to increase the quality of the matching process, which is essential for autonomous object manipulation in cluttered scenes. Fig. 6 presents the proposed closed-loop control system.

The idea of this approach is to extract two independent parallel feature streams (Maxima and Minima SIFT features) from both the model and test images and then match them to features belonging to the corresponding streams to estimate two independent affine transformations. The dissimilarity between these transformations is used as a feedback variable to observe and control the matching process. If this variable is larger than a certain threshold, one of the transformations is selected using a fuzzy controller to warp the model image. The procedure is repeated until the two transformations become similar or until one of them converges to the identity matrix.

We use a similar principle for improving the quality and the quantity of the matching process, which enhances the efficiency of the object recognition system. To close the loop, a quantitative measurement should be defined to describe the quality of the matching result and to modify the input of the image matching process for improving its output when the matching result is not accepted. The definition of this quantitative measurement is because the SIFT feature locations are efficiently detected by identifying the Maxima and Minima of the difference-of-Gaussian (DoG) scale space.

Each set of the SIFT features for the test image GF^test^ and for the model image GF^*model*^ is divided into two subsets: one for the Maxima SIFT features and the other for the Minima SIFT features.

$$\begin{array}{l} GF^{\mathrm{mod}el}=GF_{\min}^{\mathrm{mod}el}\cup GF_{\max}^{\mathrm{mod}el}\\ GF^{\text{test}}=GF_{\min}^{\text{test}}\cup GF_{\max}^{\text{test}} \end{array}$$ (8)

By matching the Maxima SIFT features with Maxima and the Minima SIFT features with Minima, two independent sets of positive matches *GM*_max_ and *GM*_min_ are obtained.

$$\begin{array}{l} GM_{\min}=match(GF_{\min}^{\mathrm{mod}el},GF_{\min}^{\text{test}})\\ GM_{\max}=match(GF_{\max}^{\mathrm{mod}el},GF_{\max}^{\text{test}}) \end{array}$$ (9)

From these sets of positive matches, two independent affine transformations (Maxima and Minima affine transformations) can be estimated using the RANSAC algorithm.

$$\begin{array}{l} T_{\min}=RANSAC(GF_{\min})\\ T_{\max}=RANSAC(GF_{\max}) \end{array}$$ (10)

The next step is to calculate the dissimilarity between the two affine transformations. Because at least three non-collinear corresponding points between two images are required to determine the affine transformation, at least three non-collinear points are required to compute the dissimilarity between two affine transformations *T*_1_ and *T*_2_. Assuming that *p*_1_ (a,a), *p*_2_ (*a*,-*a*), and *p*_3_ (-*a*,*a*), are three non-collinear points in the xy plane, where a is an arbitrary value, each of these points is mapped by each affine transformation:

$$\begin{array}{l} P_{i}^{1}=T_{1}\cdot p_{i}\\ P_{i}^{2}=T_{2}\cdot p_{i} \end{array}$$ (11)

where *i* = 1,2,3. Hence, the dissimilarity *Dis*(*T*_1_,*T*_2_) is defined as:

$$Dis(T_{1},T_{2})=\frac{1}{3}\sum_{i=1}^{3}\left(d(p_{i}^{1},p_{i}^{2})\right)$$ (12)

where *d*(*p*_1_,*p*_2_) is the Euclidian distance between two points *p*_*1*_(*x*_1_,*y*_1_) and *p*_*2*_(*x*_2_,*y*_2_) and is computed as follows:

$$d(p_{1},p_{2})=\sqrt{\left(x_{1}-x_{2})+(y_{1}-y_{2}\right)}$$ (13)

The dissimilarity between these transformations is used as a signal, indicating the matching quality. The transformations are fed back to a controller to improve the matching result. Because there is no available mathematical model for the system, a fuzzy controller is used. The dissimilarities between the identity matrix and each of the affine transformations are delivered to the fuzzy controller. The task of the controller is to select the best transformation to produce a new model image to be used in the next matching iteration as long as the termination criterion is not met. For each channel (Maxima and Minima) of the object recognition system, the error *e*_max/min_, which is computed according to equation 12, and the error derivative Δ*e*_max/min_ are chosen as inputs:

$$\begin{array}{l} e_{max/min}=Dis\left(T_{max/min},I\right)\\ \Delta e_{max/min}=e_{max/min}\left(k-1\right)-e_{max/min}\left(k\right) \end{array}$$ (14)

where I is the identity transformation given by:

$$I=\left[\begin{array}{l} 1\;0\;0\\ 0\;1\;0 \end{array}\right]$$ (15)

**Fig 7:**
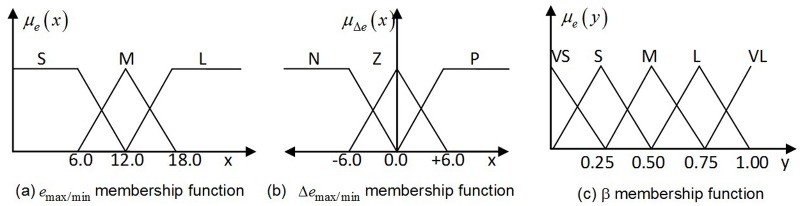
Input and output membership function.

The output is defined as a quality index, which is a real value in the range [0,1] representing how correct the corresponding affine transformation estimation is. The fuzzy controller consists of three main stages: the formation of membership functions, definition and evaluation of fuzzy rules and selection of defuzzification. In the proposed method, a triangular shape is selected as the main membership function. The range values are determined experimentally. For each input, three linguistic variables are used: S(small),M(middle),and L(large) for the error *e*_max/min_ and Z(zero),N(negative),and P(positive) for the error derivative. For each output, five linguistic variables are defined: VS(very small),S(small),M(middle),L(large),and VL(very large). The membership function for fuzzification is presented in Fig. 7. The fuzzy rules in a linguistic form are shown in Table 1. Knowledge is interpreted using IF-THEN rules, and multiple statements are joined by the AND connective. The centroid area method is used for the defuzzification processes. In this method, the resultant membership functions are developed by considering the union of the outputs of each rule, which means that the overlapping area of the fuzzy output sets is counted only once, providing additional results. The center of gravity of the shape is mathematically obtained by the following equation:

$$\bar{x}=\frac{\int_{x_{s}}^{x_{e}}x\cdot \mu_{\beta }\left(x\right)dx}{\int_{x_{s}}^{x_{e}}\mu_{\beta }\left(x\right)dx}$$ (16)

The proposed controller is based on fuzzy expert rules and uses the triangular membership functions for fuzzification, max/min operators for inference, and centroid area method for the defuzzification processes. The method has been verified through several experiments on the object recognition system. The obtained results indicate that the proposed approach is very promising.

**Table 1:** Fuzzy-expert rules in linguistic form.

Rule 1	IF (N is L) AND (e is S) AND (Δe is N)	THEN (β is M)
**Rule 2**	IF (N is L) AND (e is S) AND (Δe is N)	THEN (β is L)
**Rule 3**	IF (N is L) AND (e is S) AND (Δe is N)	THEN (β is VL)
**Rule 4**	IF (N is L) AND (e is M) AND (Δe is N)	THEN (β is S)
**Rule 5**	IF (N is L) AND (e is M) AND (Δe is N)	THEN (β is M)
**Rule 6**	IF (N is L) AND (e is M) AND (Δe is N)	THEN (β is L)
**Rule 7**	IF (N is L) AND (e is L) AND (Δe is N)	THEN (β is VS)
**Rule 8**	IF (N is L) AND (e is L) AND (Δe is N)	THEN (β is S)
**Rule 9**	IF (N is L) AND (e is L) AND (Δe is N)	THEN (β is M)

## Results

**Fig 8:**
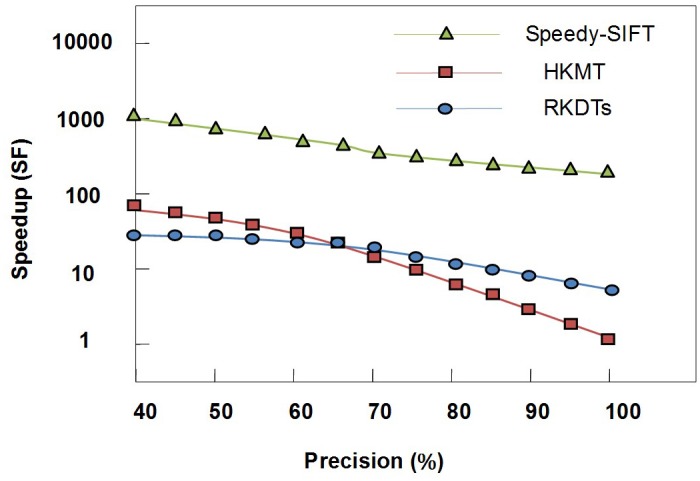
Trade-off between the matching speedup and matching precision.

To evaluate the performance of the proposed method, several experiments were conducted on different pairs of images from a standard LEAR image database [32] and from real world images. The first experiment was performed to investigate the difference between the original SIFT algorithm and the proposed optimized SIFT algorithm. The performance of the improved SIFT algorithm was compared with the performance of two algorithms: HKMT and RKDTs. The comparisons were performed using the fast library for approximate nearest neighbors (FLANN). In the experiment, SIFT features were extracted from images in the LEAR database; subsequently, each of the two corresponding images were matched using the HKMT, RKDT and speedy SIFT algorithms under different degrees of precision. The matching step performs tradeoffs between the matching speedup and matching accuracy, and the experimental results are shown in Fig. 8. The precision degree is defined as the ratio between the number of correct matches returned using the considered algorithms and the exhaustive search, whereas the speedup factor is defined as the ratio between the exhaustive matching time and the matching time of the corresponding algorithm. As shown in Fig. 8, the speedy SIFT algorithm outperforms the other two considered algorithms in speeding up feature matching for all precision degrees. For a precision of approximately 95%, the speedy SIFT algorithm obtains a speedup factor of approximately 1250.

**Fig 9:**
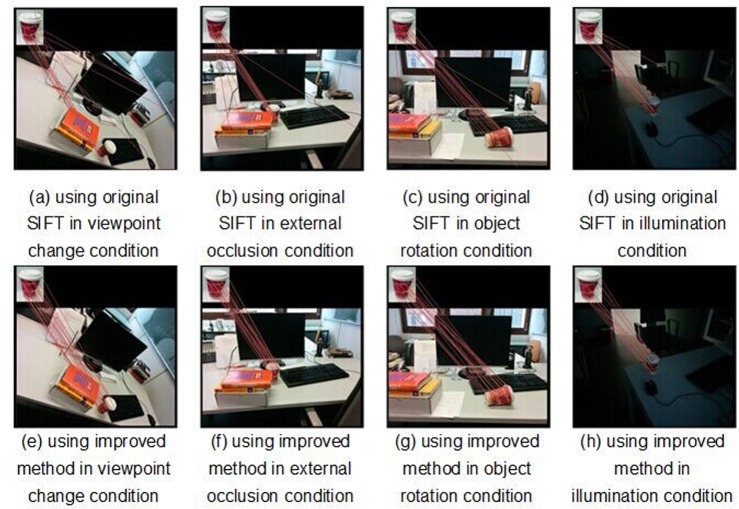
The result of the object recognition based on the original SIFT algorithm and the improved method.

In the second experiment, the proposed object recognition system was compared with the original SIFT-based method under the cluttered scene condition. A variety of images photographed by a regular digital camera were used in the experiments. The input of the system is a model image where only a single target object is available, and the test image includes the target object captured under the cluttered scene condition. The recognition progress was performed using an Intel Core2 2.6-GHz processor with images of size 1024×768 pixels. The comparison results for the coffee cup recognition in the cluttered scene are illustrated in Fig. 9. Tables 2 and 3 demonstrate the comparison of the SIFT feature matching results between the original SIFT algorithm and the improved SIFT algorithm. In Table 2, a comparison is performed to show an improvement in the robustness of the feature matching process. The number of correct SIFT features is significantly increased. Table 3 presents the computational matching time of the proposed SIFT approach and of the original SIFT approach. Compared with the original SIFT algorithm, a 40% reduction in processing time was achieved. Thus, the appearance of objects in the test images is different from their appearance in model images because of different conditions, such as illumination during image acquisition, viewpoint, partial occlusion, rotation, and illumination conditions. The advantage of the proposed recognition technique over the original SIFT matching technique is evident.

**Table 2:** Comparison of the Feature Number for the Improved SIFT and the Original SIFT.

Cluttered Scene	Feature number of original SIFT algorithm	Feature number of improved SIFT algorithm
**Viewpoint change**	73	117
**External occlusion**	77	123
**Object rotation**	68	102
**Illumination**	70	105

**Table 3:** Comparison of the computation speed for the Improved SIFT and the Original SIFT.

Cluttered Scene	Computation speed of original SIFT algorithm (s)	Computation speed of improved SIFT algorithm (s)
**Viewpoint change**	0.186	0.111
**External occlusion**	0.192	0.120
**Object rotation**	0.170	0.103
**Illumination**	0.220	0.132

## Discussion

Most applications of object recognition, such as face recognition systems and robotic vision, require efficient performance. Conventional methods are time consuming, and their performance always drops significantly during partial occlusions, large pose variations, and extreme ambient illumination conditions. Thus, this paper proposed a method for fast object recognition in cluttered scenes based on an improved SIFT algorithm and a fuzzy closed-loop control strategy. The proposed improved method is highly distinctive and significantly speeds up object feature matching by dividing the SIFT features into several clusters, restricting the matching tactics based on the scale factor, and decreasing the portion of outliers among positive matches, which leads an improvement in the robustness of the object recognition system in a cluttered background.

Possible directions for future work on the proposed methods are as follows. First, the original SIFT algorithm contains some easy but computationally intensive operations, such as Gaussian filtering and the detection of scale-space extremes, and the proposed fast SIFT algorithm is based on dividing features into several subsets. Therefore, the feature matching process can be parallelized so that it can be adapted to parallel computation and can be implemented with a hardware pipeline in the field programmable gate array (FPGA). Achieving an on-chip architecture for the SIFT algorithm would be novel way to obtain an on-chip hardware and software co-design, which provides flexibility to the users to customize the SIFT feature descriptors according to the needs of the object recognition application. Second, another main application of the fuzzy closed-loop control strategy is camera calibration. Camera calibration is the estimation of a camera’s intrinsic, extrinsic, and lens-distortion parameters. Typical uses of a calibrated camera are for correcting optical distortion artifacts, estimating the distance of an object from a camera, measuring the size of objects in an image, and constructing 3D views for augmented reality systems. Camera calibration is an important and potential application in computer vision tasks.
